# An Acute Hepatic Hydrothorax Causing Tension

**DOI:** 10.7759/cureus.71543

**Published:** 2024-10-15

**Authors:** Dina R Assali, Austin Richardson, Andrew Kalnow

**Affiliations:** 1 Emergency Medicine, OhioHealth Doctors Hospital, Columbus, USA; 2 Pulmonology and Critical Care, OhioHealth Doctors Hospital, Columbus, USA

**Keywords:** ascites, cirrhosis, hypoxemia, tension hydrothorax, thoracentesis

## Abstract

Patients with advanced cirrhosis are at risk for numerous complications, including hepatic hydrothorax. Hepatic hydrothorax most commonly occurs in the right pleural space but less commonly can present on the left hemithorax. The exact pathophysiology is not fully understood, but there are several schools of thought for right-sided effusions. In this case report, we discuss the findings of a 76-year-old female who experienced a left-sided hydrothorax likely stemming from her underlying liver disease which created tension pathology in an otherwise stable patient. Interestingly, this accumulation occurred over a couple of weeks despite optimal medical management. A thoracentesis confirmed ascitic fluid. In the final stages of hepatic failure, pleural effusions are typically found and are associated with a higher mortality rate.

## Introduction

Etiologies of pleural effusions vary and can include heart failure, liver disease, pneumonia, and/or cancer [1]. Fluid accumulation between the parietal and visceral space of the lung can lead to significant complications. Often, these effusions can cause hypoxia, dyspnea, and/or overt respiratory failure. Rarely, hepatic hydrothorax can be seen, which is defined as a pleural effusion of greater than 500 mL in patients with cirrhosis without any coexisting cardiopulmonary disease [2]. Moreover, only 5-10% of cirrhotic patients go on to develop hepatic hydrothoraces [3], making these types of effusions even less common. Fluid accumulation can continue to exceed the pleural space leading to tension physiology [4], which typically occurs in the right hemithorax. A tension hydrothorax on the left hemithorax has rarely been documented in the literature. In this case study, we report a left-sided tension hepatic hydrothorax creating a right-sided mediastinal shift with tracheal deviation in the setting of cirrhosis secondary to primary sclerosing cholangitis.

## Case presentation

A 76-year-old female with a medical history of primary sclerosing cholangitis, non-alcoholic steatohepatitis cirrhosis complicated by ascites, hypothyroidism, non-insulin-dependent diabetes mellitus 2, and depression presented to the emergency department (ED) for shortness of breath and newly found hypoxia requiring 3-5 L on nasal cannula (NC). She was compliant with her spironolactone, torsemide, rifaximin, lactulose, ursodiol, and midodrine and was generally functional at home, not requiring assistance or supplemental oxygen for her day-to-day activities. Before she arrived at the ED, she had undergone an outpatient procedure with interventional radiology for placement of an Aspira-tunneled abdominal drain for at-home drainage of her ascitic fluid. The goal of this drain was to allow her to be more comfortable at home, as she was in the process of establishing hospice. Before the procedure, during her triage, she was found to be hypoxic at 86% on room air with a respiratory rate of 21 breaths/minute, blood pressure of 117/72 mmHg, heart rate of 84 beats/minute, and a temperature of 97.1°F. After the successful placement of the abdominal catheter, she was referred to the ED for a workup of the hypoxia. There, her vitals were a blood pressure of 113/68 mmHg, heart rate of 83 beats/minute, respiratory rate of 16 breaths/minute with an SpO_2_ of 86-88%, and temperature of 98.2°F.

**Figure 1:**
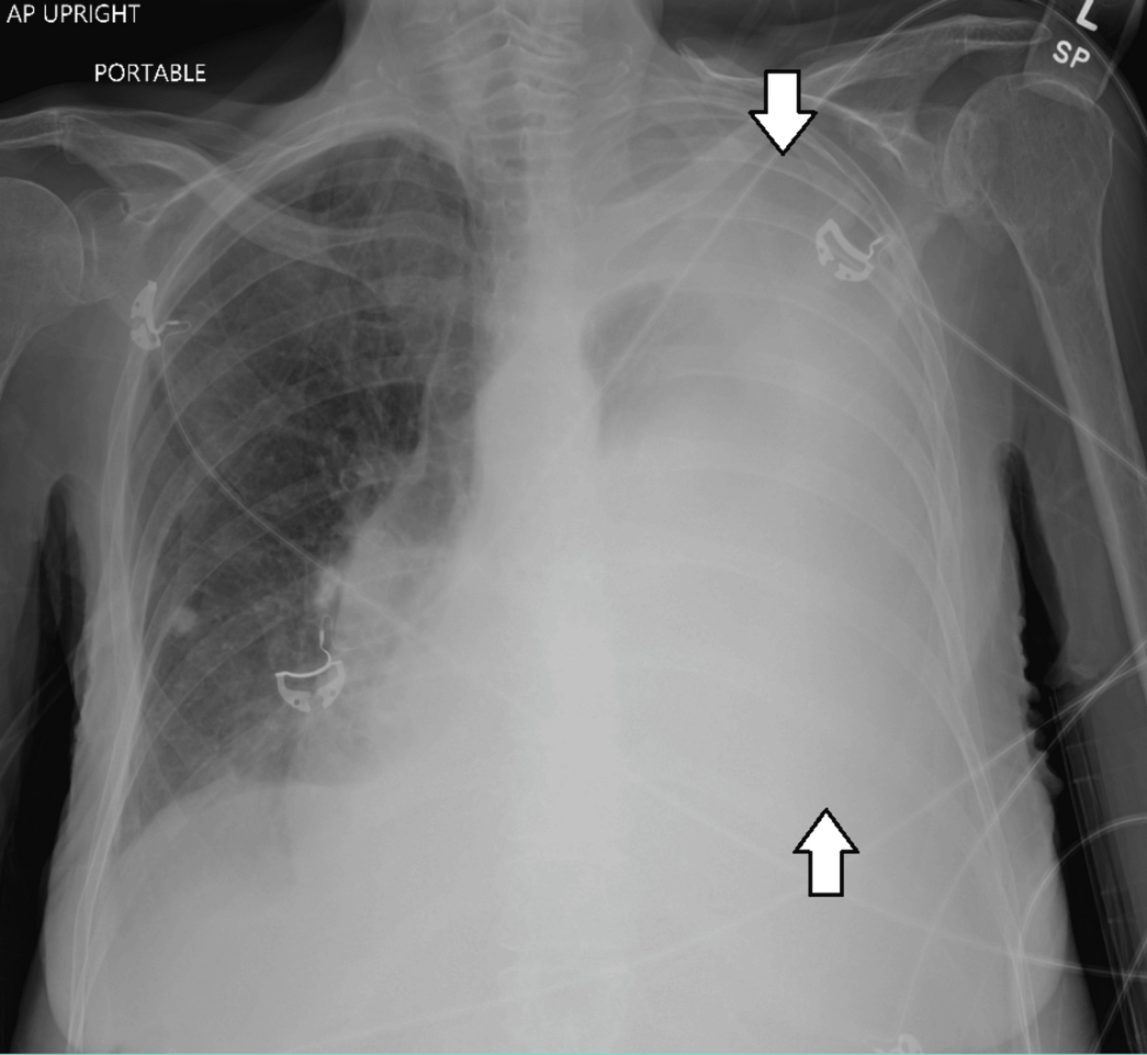
Portable anteroposterior chest X-ray view on arrival to the emergency department before thoracentesis.

**Figure 2:**
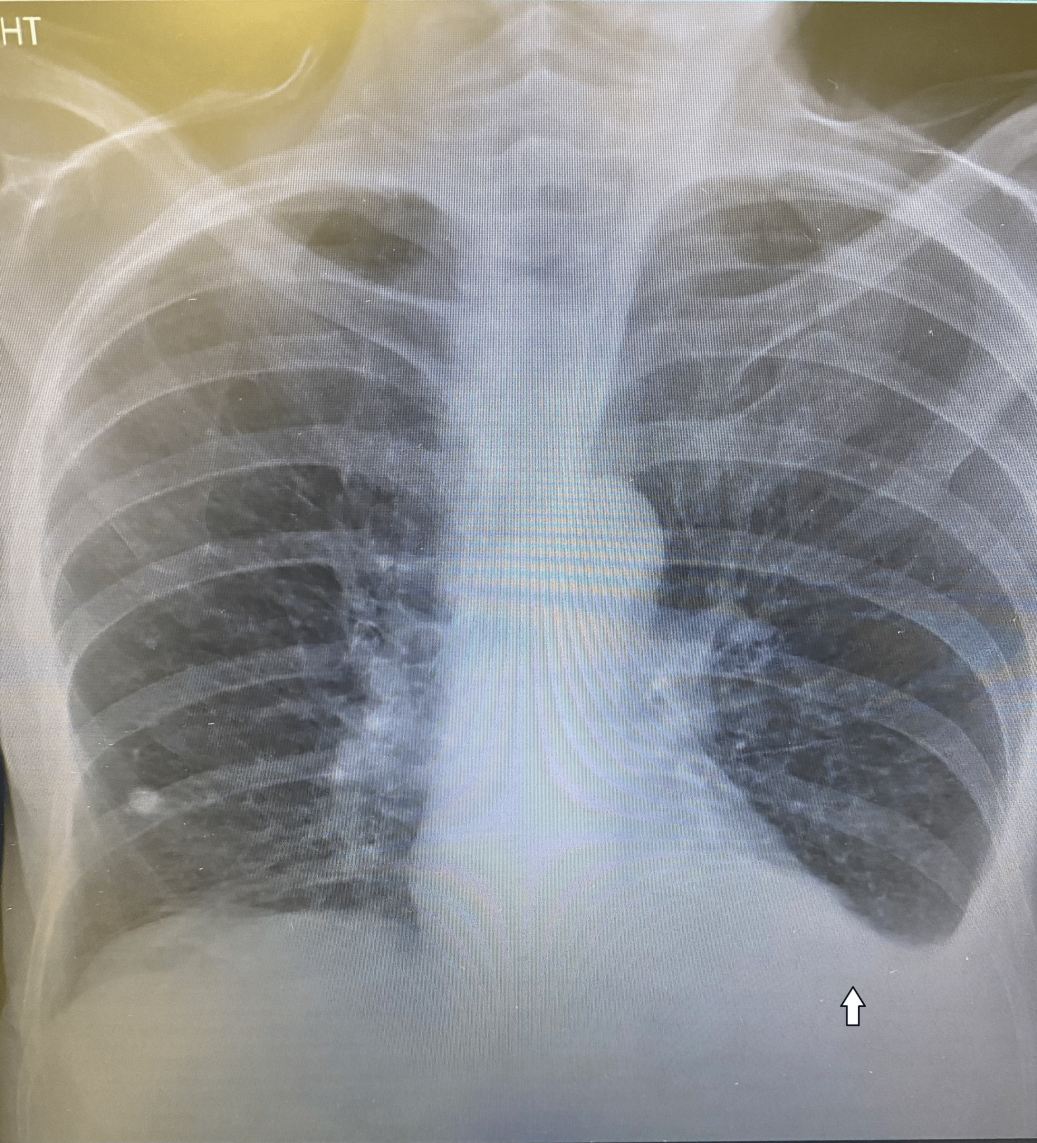
Anteroposterior chest X-ray 19 days before arrival at the emergency department.

**Figure 3:**
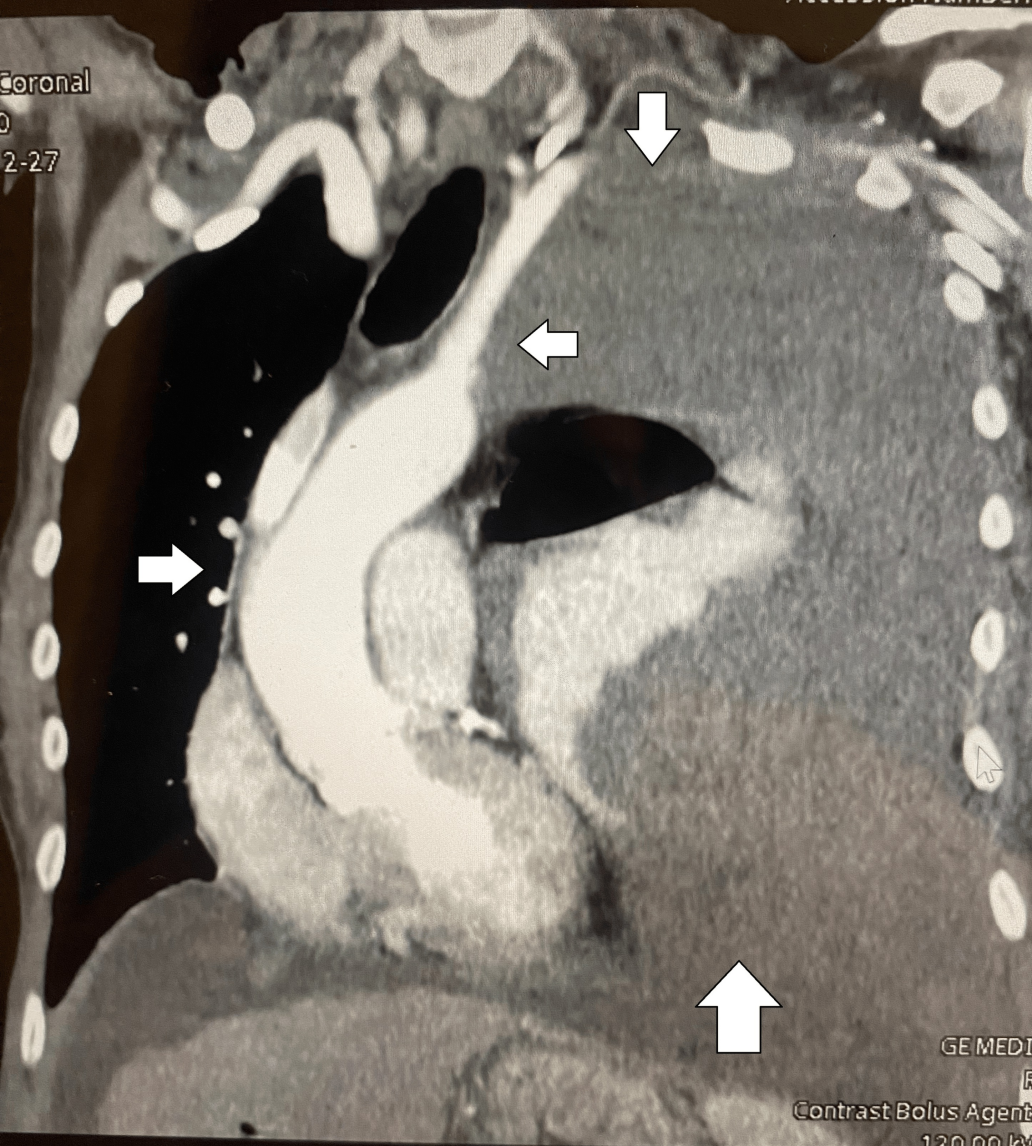
CT of the thorax with contrast sagittal view displaying tracheal deviation before thoracentesis.

**Figure 4:**
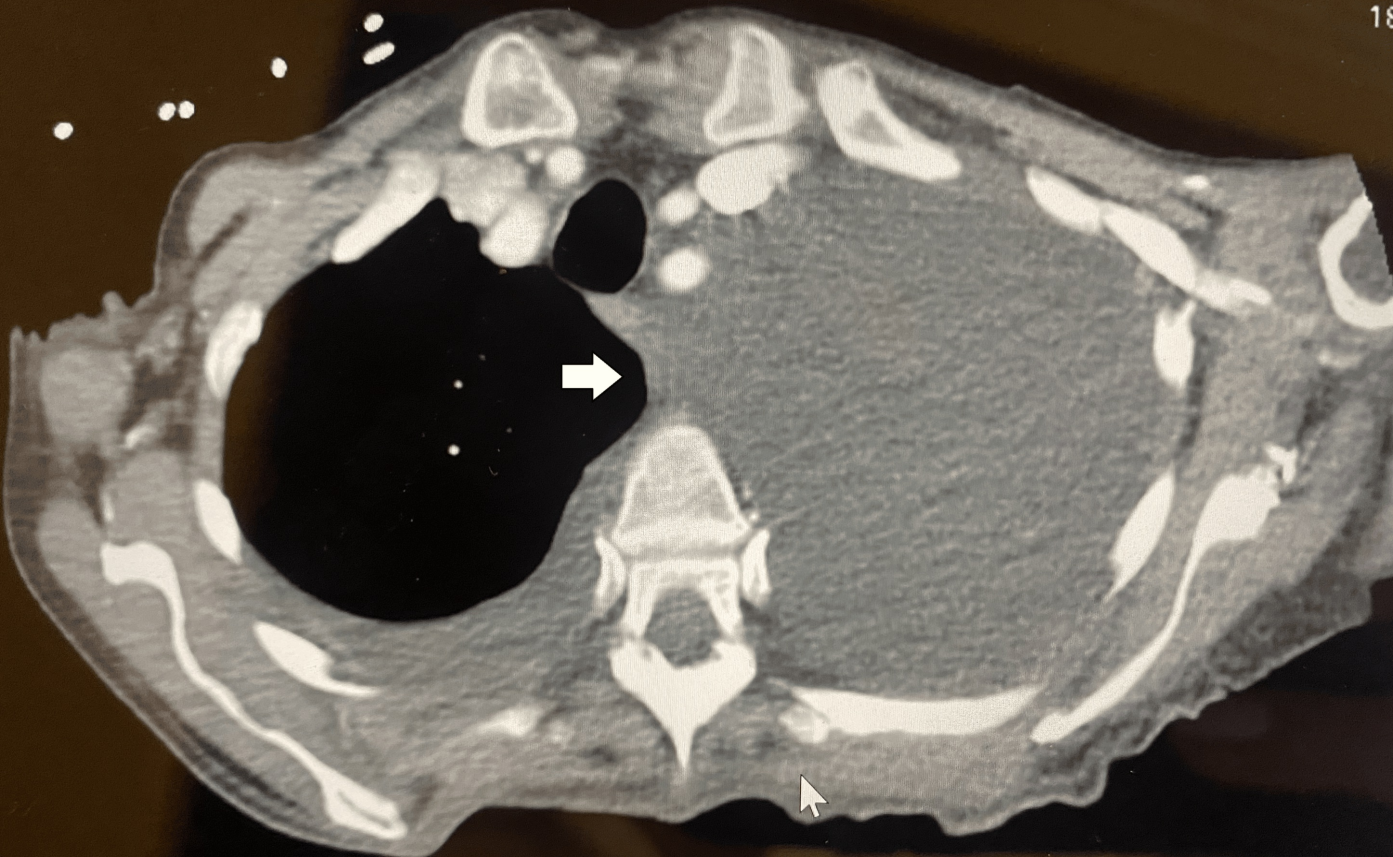
CT of the thorax with contrast axial view with complete opacification of the left lung before thoracentesis.

On physical examination, she had increased egophony present on her left hemithorax with increased work of breathing but was not toxic-appearing. A chest X-ray confirmed a large left pleural effusion with a near-complete white-out of the left lung (Figure 1). This was a major change from her most recent chest X-ray which showed minimal left-sided pleural effusion (Figure 2). A follow-up computed tomography scan of the thorax with contrast showed a near-complete collapse of the left upper lobe with partial collapse of the left lower lobe in addition to the mediastinal shift and tracheal deviation (Figures 3, 4).

**Figure 5:**
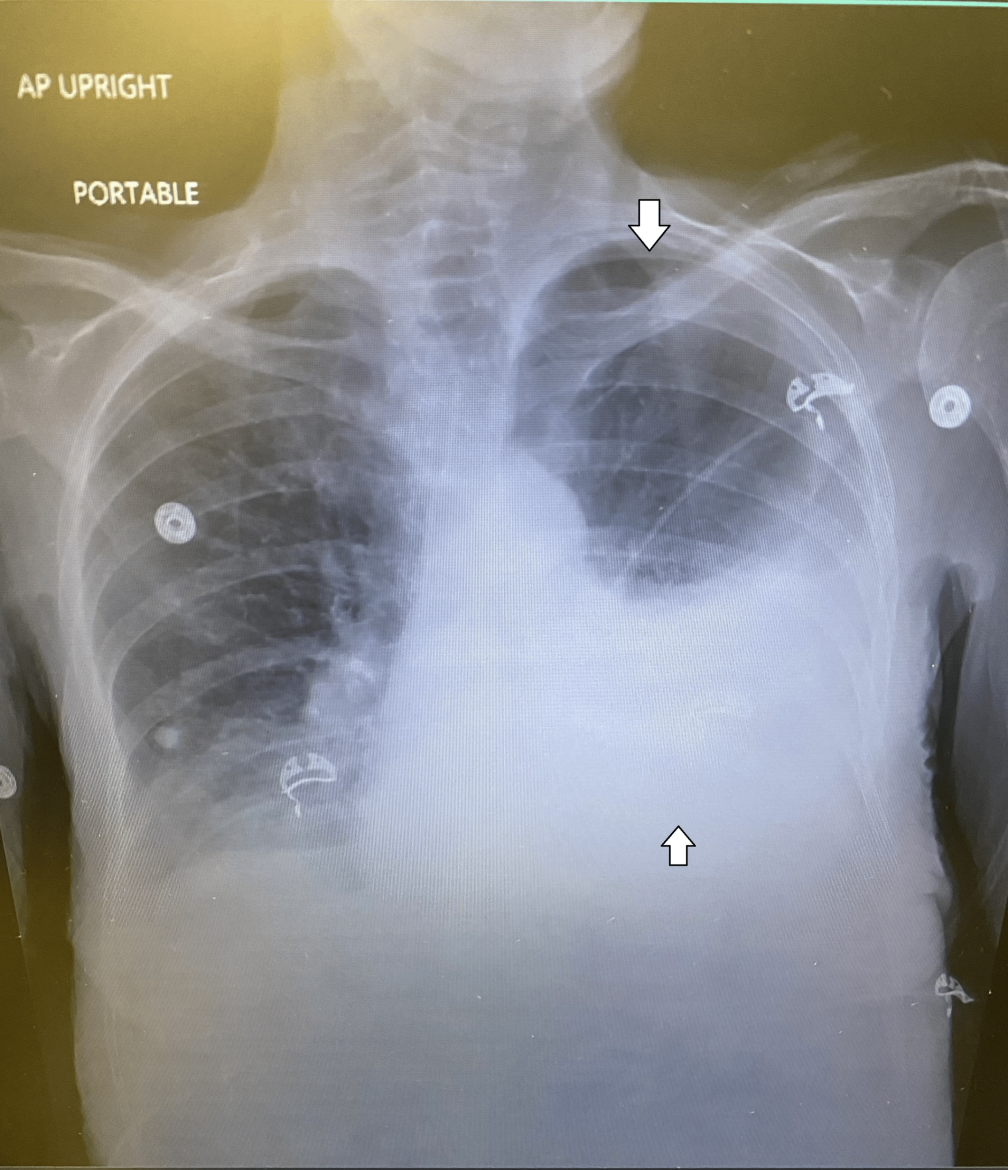
Portable chest X-ray view post-thoracentesis.

Consequently, the patient was admitted to the hospital and underwent an urgent thoracentesis on the floor. Approximately 1 L of clear-yellow fluid was drained via an ultrasound-guided technique at the bedside but was aborted due to excessive coughing and discomfort before complete drainage. Afterward, her oxygen requirements were reduced, although the patient still had the effusion on a repeat chest X-ray (Figure 5); however, the resolution of tracheal deviation was seen with complete re-expansion. Fluid analysis was suggestive of ascitic fluid without malignant cells, confirming hepatic hydrothorax (Table 1). Unfortunately, the patient was lost to follow-up as she left the hospital before completing care. 

**Table 1:** Thoracentesis findings.

	Thoracentesis value	Serum value
Protein	2.9 g/dL	-
Glucose	105 mg/dL	-
Lactate dehydrogenase	48 U/L	112 U/L (100–250 U/L)
Albumin	1.7 g/dL	2.4 g/dL (3.2–5.2 g/dL)
Cytology	Negative for malignant cells	-
Cell color	Yellow appearance	-
Cell count (red blood cells)	Hazy red blood cells: 22 (0 for reference)	-
Cell count (white blood cells)	119 (0–1,000 µL)	-
Cell count (neutrophils)	10 (0–25%)	-
Cell count (lymphocytes)	75 (0 for reference)	-
Cell count (monocytes)	10 (0 for reference)	-
Cell count (meso/macro)	5 (0 for reference)	-

## Discussion

There are case reports describing cirrhosis-induced tension hydrothoraces. What differentiates this case study from others is that, first, our patient was an otherwise stable patient with tension pathology, although decompensation was inevitable. Only about 10% of pleural effusions make up massive pleural effusions, wherein they are considered to occupy greater than two-thirds of the hemithorax [5,6], and 67% of which are considered to be from malignant pulmonary etiology [7]. Second, this effusion was present on the left hemithorax and was not from primary malignancy. Studies have shown that pleural effusions from ascites tend to develop more frequently on the right in comparison to the left due to the positive intra-abdominal pressure and the presence of diaphragmatic defects [2,8]. There are believed to be small defects measuring less than 1 cm in the tendinous portion of the diaphragm [2], allowing ascitic fluid to move along a negative pressure gradient from the abdomen into the pleural space [9,10]. Other schools of thought suggest that the leakage of fluid from the right thoracic duct occurs through an increase in azygous vein pressure caused by portal hypertension [2]. Third, it is infrequent for patients to display hepatic hydrothorax in the setting of medical management, including sodium restriction and diuretic therapy [3], as was seen in our patient who was compliant with her medications. In patients needing escalation of care for mild-to-moderate pleural effusion accumulation, thoracenteses can be done therapeutically. Contrary to common belief, chest tubes are not indicated in these hydrothorax cases [3,11]. Studies have shown that chest tube placement can prolong hospital stay, increase mortality, and worsen complications, including infection, bleeding, or even flash pulmonary edema due to rapid fluid removal [3]. A chest tube or pigtail allowing for continuous drainage is not the answer for such patients. With repeat accumulation, a transjugular intrahepatic portosystemic shunt, liver transplant, and/or surgical repair of diaphragmatic defects have been done to provide relief [8,9]. Unfortunately, the finding of hepatic hydrothorax is associated with a greater mortality rate with approximately half of the patients dying within one year of presentation, according to a study conducted by Badillo and Rockey in 2014 [9]. Tension hydrothorax is of great concern when it is caused by ascitic fluid due to the likelihood and frequency of re-accumulation from the ongoing nature of ascites and needs urgent thoracentesis.

## Conclusions

When complete opacification is seen on chest X-ray in the setting of hypoxemia similar to our patient’s presentation, following up with CT imaging to evaluate for tension pathology is necessary for appropriate and timely management. In this case report, an urgent thoracentesis was done to relieve tension from fluid accumulation in an otherwise stable patient. Moving forward, however, if the patient is hemodynamically unstable to include supplemental oxygen requirements, an emergent thoracentesis is necessary while in the ED. It is important to note that according to prior studies, placing a chest tube in patients with pleural effusions secondary to liver disease may actually increase mortality and worsen complications.
